# A new 2D-based method for myocardial velocity strain and strain rate quantification in a normal adult and paediatric population: assessment of reference values

**DOI:** 10.1186/1476-7120-7-8

**Published:** 2009-02-13

**Authors:** C Bussadori, A Moreo, M Di Donato, B De Chiara, D Negura, E Dall'Aglio, E Lobiati, M Chessa, C Arcidiacono, JS Dua, F Mauri, M Carminati

**Affiliations:** 1 Pediatric Cardiology and Adult with Congenital Heart Disease Department, IRCCS San Donato Hospital, Milan, Italy; 2 Cardiology Department, Niguarda Ca' Granda Hospital, Milan, Italy; 3 Adult Clinical Researches Cardiac Surgery Department, IRCCS San Donato Hospital, Milan, Italy

## Abstract

**Background:**

Recent advances in technology have provided the opportunity for off-line analysis of digital video-clips of two-dimensional (2-D) echocardiographic images.

Commercially available software that follows the motion of cardiac structures during cardiac cycle computes both regional and global velocity, strain, and strain rate (SR).

The present study aims to evaluate the clinical applicability of the software based on the tracking algorithm feature (studied for cardiology purposes) and to derive the reference values for longitudinal and circumferential strain and SR of the left ventricle in a normal population of children and young adults.

**Methods:**

45 healthy volunteers (30 adults: 19 male, 11 female, mean age 37 ± 6 years; 15 children: 8 male, 7 female, mean age 8 ± 2 years) underwent transthoracic echocardiographic examination; 2D cine-loops recordings of apical 4-four 4-chamber (4C) and 2-chamber (2C) views and short axis views were stored for off-line analysis.

Computer analyses were performed using specific software relying on the algorithm of optical flow analysis, specifically designed to track the endocardial border, installed on a Windows™ based computer workstation. Inter and intra-observer variability was assessed.

**Results:**

The feasibility of measurements obtained with tissue tracking system was higher in apical view (100% for systolic events; 64% for diastolic events) than in short axis view (70% for systolic events; 52% for diastolic events). Longitudinal systolic velocity decreased from base to apex in all subjects (5.22 ± 1.01 vs. 1.20 ± 0.88; p < 0.0001). Longitudinal strain and SR significantly increased from base to apex in all subjects (-12.95 ± 6.79 vs. -14.87 ± 6.78; p = 0.002; -0.72 ± 0.39 vs. -0.94 ± 0.48, p = 0.0001, respectively). Similarly, circumferential strain and SR increased from base to apex (-21.32 ± 5.15 vs. -27.02 ± 5.88, p = 0.002; -1.51 ± 0.37 vs. -1.95 ± 0.57, p = 0.003, respectively).

Values of global systolic SR, both longitudinal and circumferential, were significantly higher in children than in adults (-1.3 ± 0.2, vs. -1.11 ± 0.2, p = 0.006; -1.9 ± 0.6 vs. -1.6 ± 0.5, p = 0.0265, respectively). No significant differences in longitudinal and circumferential systolic velocities were identified for any segment when comparing adults with children.

**Conclusion:**

This 2D based tissue tracking system used for computation is reliable and applicable in adults and children particularly for systolic events. Measured with this technology, we have established reference values for myocardial velocity, Strain and SR for both young adults and children.

## Background

Accurate assessment of global and regional left ventricular function is essential for the evaluation and management of patients with heart disease. Recent developments have improved cardiac function quantification and it seems that both magnitude and temporal sequence of tissue deformation can provide additional information in known or suspected heart disease [1-3].

Tissue Doppler Imaging (TDI) has been extensively applied in assessing and quantifying regional myocardial contractility [4-6] and validated against sonomicrometry and magnetic resonance imaging (MRI) [7]. Furthermore, simultaneous recording of myocardial velocities allows estimation of Strain (ε) and Strain Rate (SR). In fact, using TDI based technology, SR is calculated as (V1–V2)/L where L is the distance between the two points whose velocities are measured and Strain (ε) is obtained by temporal integration of SR. Strain provides a dimensionless measure of the total deformation the myocardium undergoes during contraction and is expressed as percentage while SR is expressed in s^-1 ^[8].

However, TDI being a Doppler based technique, its routine clinical use is limited by technical issues such as angle, signal noise and measure variability. When the angle between the velocity direction and the ultrasound beam is > 20°, the real velocity is underestimated. Since a correct alignment is not always possible, due to ventricular geometry, TDI derived measures will lose validity especially at the apical segments [9]. Recently, improvements in 2D echocardiographic image resolution have enabled detection of tissue pixels and tracking of these acoustic markers from frame to frame. The tissue velocity is estimated from the local frame-to-frame displacement; the automatic evaluation of the velocity at a point is determined by comparison of the displacement of the image data around that point in two consecutive frames. These methods have been used, in several different formulations, in many research fields and fall in the category known as Optical Flow [8], commonly referred as Speckle Tracking in ultrasound imaging.

Several 2D tissue tracking techniques are currently available and their difference is based on the type of algorithm employed; the first (and more investigated) method is known as "speckle tracking" [1] and the second one is known as "feature tracking" [10]. In this latter method the software is based on a dedicated algorithm, that follows frame by frame, the endocardial border traced by the operator; actually this processing system is based on a mono-dimensional technology which seems to be more accurate with respect to 2D processing. The present study aims to evaluate the clinical applicability of a specifically designed tissue tracking software: XStrain™ (Esaote, Florence) based on an original and patented concept [European Patent Specification: EP1520517] and to derive reference values for longitudinal and circumferential velocity, ε and SR of the left ventricle in a population of normal children and young adults.

## Methods

### Study population

Forty-five healthy volunteers (30 adults: 19 male, 11 female, mean age 37 ± 6 years; 15 children: 8 male, 7 female, mean age 8 ± 2 years) were included in the study. Informed written consent and appropriate ethics approval were obtained. All subjects underwent clinical examination, 12-lead ECG recordings and systemic blood pressure recording. Subsequently, they underwent a complete transthoracic echocardiographic examination in left lateral decubitus. The exclusion criteria were: history of hypertension, diabetes, coronary artery disease, myocardial infarction, stroke, congenital heart disease, acquired valve disease, atrial fibrillation or congestive heart failure.

### Echocardiographic study

Echocardiographic exams were performed with all subjects positioned in the left lateral decubitus, by the same operator (CB) using a MyLab50 echo machine (Esaote, Florence) equipped with a multi-frequency 2.5–3.5 MHz transducer. 2D harmonic image cine-loops recordings (video clips) of apical 4C and 2C views and short axis views at mitral, papillary muscles and apical levels, with good quality ECG signal and a frame rate between 40–64 fps were acquired and stored for off-line analysis. Off-line computer based analysis of the video clips stored on the Esaote Mylab 50 were performed using XStrain™ software (Esaote, Florence) installed on a Windows™ based computer work station. Relying on the algorithm of optical flow analysis specifically designed to track the endocardial border, XStrain™ is a dedicated software that derives longitudinal and circumferential myocardial velocity, ε and SR from the digitized 2D video clips. The endocardial border, drawn by the operator on an arbitrary single frame, is identified as a sequence of points. It is then automatically followed frame by frame by searching (in the greyscale pattern) for each single point, in the neighbourhood of maximum likelihood. For apical views an adaptive control is performed by the system; the control is based on the spatial coherence of the points with respect to the points fixed by the operator (mitral annulus and apex).

**Figure 1:**
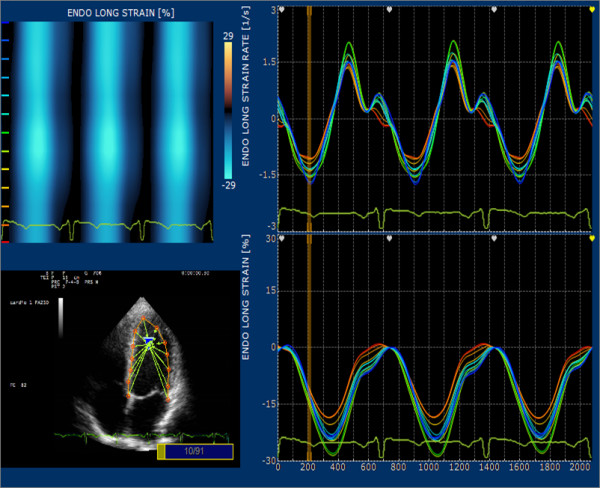
**Graph with parts of the processing result of an apical four chambers view**. The vectors show the direction of displacement of any point selected on top left, on upper left color scale analysis of systolic strain, on the right side curves for Strain and SR any selected points.

For each point, the velocities are calculated and displayed both as vectors superimposed on the 2D images and as graphs plotted against time; ε and SR displayed for each point are deformation and deformation rate of the segment whose extreme points are the previous and the subsequent points with respect to the point analyzed (Figure 1). Since point selection is the most important issue in post processing and requires new skills even for experienced cardiologist, inter and intra-observer variability was tested. To this purpose longitudinal Strain and SR were measured in 10 subjects for all six segments, blindly on two separate days by two experienced operators (CB and ED).

**Figure 2:**
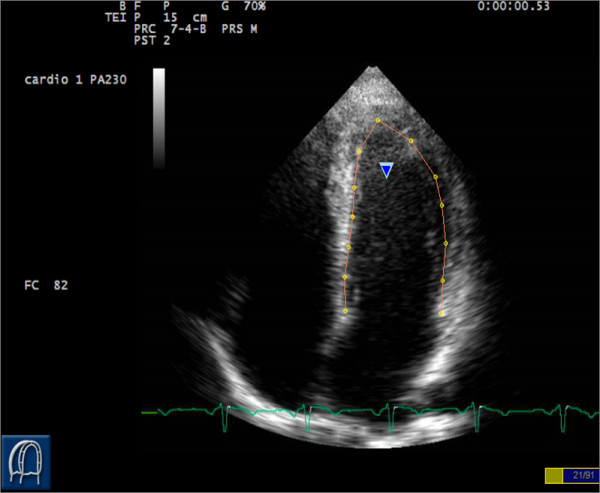
**Points positioning**. Selection of 13 equidistant points dividing the left ventricle in segments according to a 6 segments model.

For each patient, clip with the best border was chosen and processed as follows: a starting frame was chosen, usually in end diastole when endocardial border is better visible (but this is not mandatory since the algorithm is designed to follow points tracked in any frame). Border tracking of the left ventricle was manually traced by an experienced operator in the recorded clips. In apical 4C and 2C views a total of 13 equidistant points were tracked in order to obtain a subdivision in 6 segments for each view. Points were tracked starting from the septal side of the mitral annulus in the apical 4C view (Figure 2) and three points for each segment were tracked, with the first of each segment being the last of the previous one (except for the first point of the basal septal segment). In the apical 2C view-points are tracked starting from the mitral annulus at the level of the inferior wall.

**Figure 3:**
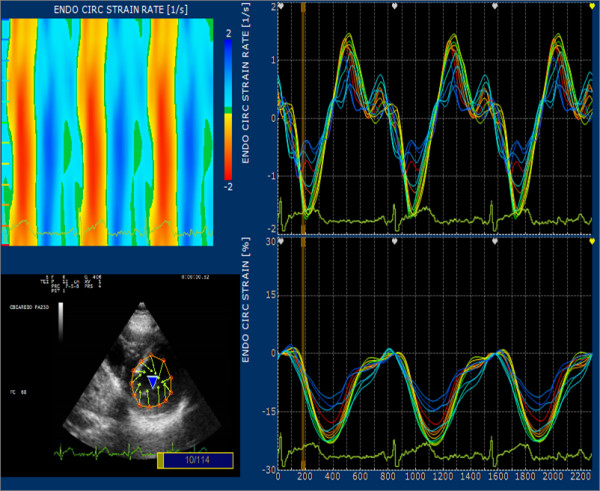
**Graph with parts of the processing result of a short axis view at mitral level**. After selection of 12 equidistant point dividing the left ventricle in segments according to a 6 segments model; on left upper the system reports color analysis of circumferential SR and the curve report the values for circumferential Strain and SR for any point selected.

In the short axis at the mitral valve level (Figure 3) the points were tracked from the postero-medial commissure of the mitral valve, using the same method as that for the apical views (last point of a segment is the first of the following). In short axis at the papillary muscle level, starting point was at the postero-medial papillary muscle, and point 8 and 12 were positioned in the internal part of the papillary muscle, at the same level as that of the other dots, in order to follow the same group of myocardial fibres. At the apex, since there are no cardiac anatomical landmarks, the apical short axis was arbitrarily divided in four quadrants drawing two perpendicular lines and positioning the first point aligned with the first point of the previous section.

Velocity, ε, and strain rate graphics were automatically obtained and quantitative data for different parameters were exported by the system into an Excel^® ^spreadsheet.

### Statistical analysis

Inter and intra-observer variability was tested using concordance correlation coefficient; precision with Pearson correlation coefficient (r) and with *C*_b _as a bias correction factor to measure accuracy [11,12]. Differences between groups or variables were compared using Student's unpaired t-test. Statistical significance was set at a p value < 0.05. The Statistical Package for Social Science (SPSS 13.0 for Microsoft Windows) was used.

## Results

### Feasibility

The feasibility of obtaining measurements with border tracking system, evaluated in all the 225 stored cine-loops, was higher in the apical long axis view (100% for systolic and 64% for diastolic events) than in the short axis view (70% for systolic and 52% for diastolic events).

### Inter and Intra-observer variability

Inter-observer variability between two experienced operators was minimum; Correlation coefficient r = 0.8958, p < 0.0001, 95% Confidence interval for r = 0.8039 to 0.9460.

Intra-observer variability for CB was r = 0.9348, 95% Confidence interval for r = 0.8968 to 0.9591, p < 0.0001. Bias correction factor C_b _(accuracy) 0.9632.

### Normal values

**Figure 4:**
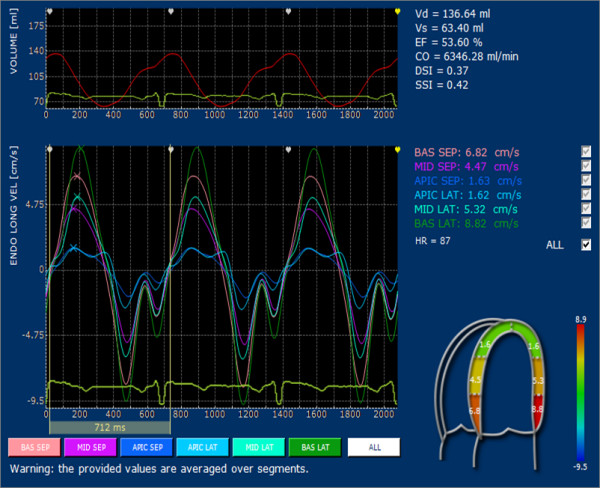
**Velocities regional analyses**. Values for longitudinal velocity averaged by segment in a normal adult are reported; notice the decreasing of velocity from base to apex both in the septum and in the lateral wall.

**Figure 5:**
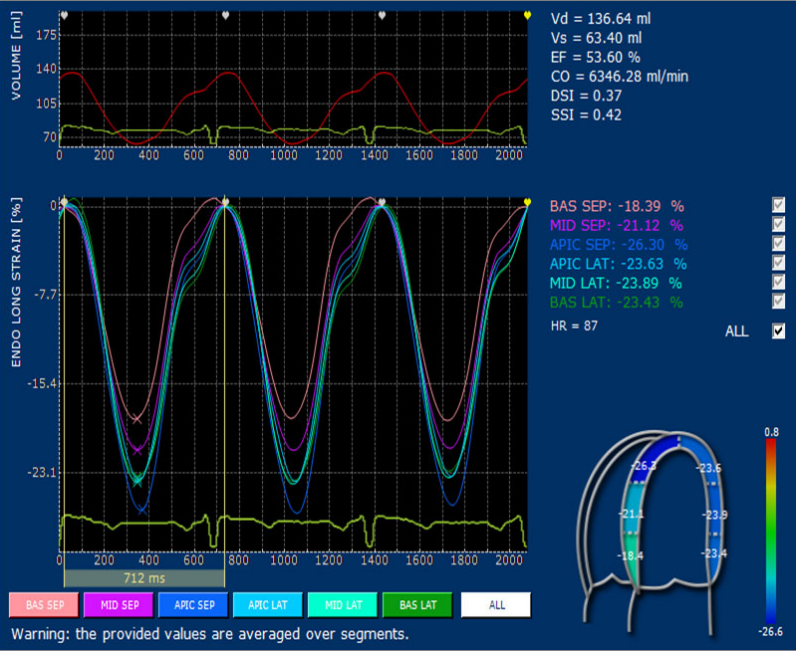
**Longitudinal strain regional analyses**. Longitudinal strain values averaged by segment in a normal adult are reported; notice the increasing of Strain from base to apex of the septal segments.

Values of the longitudinal and circumferential velocities, in systole and diastole, measured in the overall population are reported in details in Table 1. Longitudinal systolic velocities in the complete cardiac cycle, both in adults and in children, showed decreasing values from base to apex (Figure 4) similar to those previously reported in literature, based on a TDI system [13]. For diastolic velocities, children showed significantly taller E-wave peak velocities at the septal mitral annulus and at the basal septum as compared to adults, and, lower A-wave peak velocities at the septal mitral annulus in 4C segments (Table 1). Both longitudinal and circumferential ε showed a significant increase in the septal wall from base to apex (Figure 5); SR showed similar progression through base to apex (Table 2). In fact, longitudinal ε and SR significantly increased from base to apex in all subjects (-12.95 ± 6.79 vs. -14.87 ± 6.78, p = 0.002; -0.72 ± 0.39 vs. -0.94 ± 0.48, p = 0.0001, respectively). When the segmental increase from base to apex in longitudinal Strain and SR was analysed separately for age groups, the difference was strongly significant in both adults (-15.78 ± 3.63 vs. -24.00 ± 5.87, p = 0.0001 and -0.83 ± 0.21 vs. -1.44 ± 0.37, p = 0.0001) and children (-20.65 ± 4.46 vs. 25.36 ± 6.14, p = 0.0197. -1.16 ± 0.29 vs. -1.66 ± 0.40 p = 0.0004). This significant difference from base to apex was not evident at the lateral wall in children, nor in adults. Comparing children with adults, longitudinal Strain and SR were significantly higher in children at basal septum (-20.6 ± 4.4 vs. -15.8 ± 3.5, p = 0.0002; -1.2 ± 0.3 vs. -0.8 ± 0.2, p = 0.0001), at basal lateral wall (-22.9 ± 3.4 vs. -17.9 ± 5.2, p = 0.0013; -1.3 ± 0.3 vs. -1.0 ± 0.3, p = 0.0072), at mid septum (-20.9 ± 3.9 vs. -17.7 ± 4.1, p = 0.0133; -1.1 ± 0.2 vs. -0.9 ± 0.2, p = 0.0070) and at mid lateral wall (-23.3 ± 4.2 vs. -18.9 ± 4.9, p = 0.0052; -1.34 ± 0.3 vs. -1.0 ± 0.3, p = 0.0027) (Table 2)

**Table 1:** Systolic and diastolic values of the longitudinal velocities in adults and children

**Apical 4 Chambers View**								
Systolic Peak Velocity (cm/sec)								

	Sept.Mit.Ann.	Bas Sept	Mid Sept	Ap sept	Ap lat	Mid lat	Bas Lat	Mit Ann LW

Children	4.9 ± 0.6	4.3 ± 0.4	2.9 ± 0.6	1.22 ± 0.6	0.63 ± 0.5	2.3 ± 0.8	4.6 ± 0.8	5.4 ± 0.7

Adults	5.1 ± 1.2	4.4 ± 1.1	3.2 ± 0.98	1.70 ± 0.7	1.02 ± 0.97	2.9 ± 1.9	4.7 ± 1.2	5.5 ± 1.4

**P value**	0.557	0.624	0.297	**0.05**	0.147	0.264	0.831	0.939

Diastolic Peak Velocity E (cm/sec)								

Children	-7.6 ± 0.9	-6.6 ± 0.9	-3.7 ± 1.1	-1.1 ± 0.8	-0.58 ± 0.9	-3.1 ± 1.4	-6.4 ± 1.3	-8.4 ± 1.2

Adults	-6.3 ± 1.8	-5.2 ± 1.8	-3.6 ± 1.5	-1.5 ± 0.9	-0.97 ± 0.9	-3.4 ± 1.5	-6.2 ± 1.7	-7.4 ± 1.9

**P value**	**0.0075**	**0.0058**	0.8273	0.1589	0.2070	0.4230	0.7091	0.088

Diastolic Peak Velocity A (cm/sec)								

Children	-2.5 ± 1.3	-2.1 ± 1.1	-1.5 ± 0.8	-0.4 ± 0.5	-0.1 ± 0.2	-0.57 ± 0.6	-1.8 ± 1.1	-2.3 ± 1.5

Adults	-3.9 ± 1.6	-3.2 ± 1.3	-2.6 ± 1.3	-1.0 ± 1.1	-0.8 ± 0.7	1.9 ± 1.9	-2.9 ± 2.3	-3.6 ± 2.8

**P value**	**0.0201**	**0.0335**	**0.0204**	0.0936	**0.0047**	**0.0262**	0.1315	0.1461

**Table 2:** Values of longitudinal and circumferential strain and SR in adults and children.

**Apical 4 Chambers segmental mean Systolic Strain %**						
	Bas Sept	Mid Sept	Ap sept	Ap lat	Mid lat	Bas Lat

Children	-20.6 ± 4.4	-20.9 ± 3.9	-25.4 ± 6.1	-19.8 ± 5.8	-23.3 ± 4.2	-22.9 ± 3.4

Adults	-15.8 ± 3.5	-17.7 ± 4.1	-24.0 ± 0.8	19.9 ± 4.82	-18.9 ± 4.9	-17.9 ± 5.2

**P value**	**0.0002**	**0.0133**	0.4664	0.4236	**0.0052**	**0.0013**

**Global mean circumferential Systolic Strain % by sections**						
			
	Mitral	Mid Ventricular	Apex			
			
Children	-22 ± 4	-24 ± 6	-32 ± 7			
			
Adults	-21 ± 6	-22 ± 5	-27 ± 6			
			
**P value**	0.5861	0.4032	**0.05**			

Even for circumferential ε and SR there was a significant increase of the values from base to apex calculated as global values for section in all subjects (-21.23 ± 5.36 vs. -28.65 ± 6.72, p = 0.002; -1.59 ± 0.47 vs. -2.11 ± 0.69, p = 0.003, respectively). Global systolic longitudinal Strain was significantly higher in children than in adults (22.18 ± 3.06 vs. -19.05 ± 3.05 p = 0.0020). Global systolic SR, both longitudinal and circumferential, was significantly higher in children than in adults (-1.30 ± 0.20 vs. -1.07 ± 0.19, p = 0.015; -1.83 ± 0.60 vs. -1.67 ± 0.55, p = 0.04, respectively). The differences in longitudinal and circumferential Strain and SR between adults and children were more significant for circumferential strain of the apical segments.

Values of global systolic longitudinal and circumferential values of ε and strain rate are reported in Table 3.

**Table 3:** Values of global longitudinal and circumferential Strain and SR in normal adults and children.

Global Longitudinal Strain (all subjects)	-20.16 ± 3.37	Global Longitudinal SR (all subjects)	-1.16 ± 0.22
Global Longitudinal Strain Adults	-19.05 ± 3.05	Global Longitudinal SR Adults	-1.07 ± 0.19

Global Longitudinal Strain Children	-22.18 ± 3.06	Global Longitudinal SR Children	-1.30 ± 0.20

Global Circumferential Strain (all subjects)	-24.86 ± 6.76	Global Circumferential SR (all subjects)	-1.78 ± 0.58

Global Circumferential Strain Adults	-24.93 ± 7.35	Global Circumferential SR Adults	-1.67 ± 0.55

Global Circumferential Strain Children	-25.60 ± 7.08	Global Circumferential SR Children	-1.95 ± 0.60

## Discussion

TDI, it's derivatives, and 2D strain technologies suffer from an intrinsic limitation i.e. they have been put to clinical use long before their value has been demonstrated in various clinical settings. Currently, we can only make intra-population comparisons to assess changes induced by treatments that act on strain, strain rate and torsion like resynchronization therapy, drug therapy or surgery. In the near future we will have data on the clinical impact of such measurements and their clinical meaning. These measures actually assess cardiac mechanics and changes induced by loading conditions that are already well known from physiology and pathology point. But their great advantage, once technical accuracy has been achieved, is that they are easily and non-invasively applicable to large cardiac disease population.

The 2D Strain tissue tracking method described as "Feature Tracking" has previously been employed in paediatric [14] and adult cardiology [10,15-17] but to our knowledge, a complete derivation of reference values for longitudinal and circumferential parameters has not yet been published.

The processing system used in this study is based on a mono-dimensional technology; this seems to be a more accurate system and this hypothesis has been verified in this study. The "Feature Tracking" system is highly reliable for systolic events and provides data for apical and short axis regions that TDI is unable to provide. This new 2D tissue tracking technology overcomes this limitation by analyzing displacement of all the points tracked in two spatial dimensions (along the endocardial border and perpendicular to it). This has the advantage of offering a correct strain computation even along curved segments.

Limitations to our study are mostly software related and the fact that the analysis is based on 2D video clips. Any 2D based system that relies on tissue tracking needs good quality images (as young and cooperative subjects can offer); however, in clinical practice it may be more difficult to obtain a similar level of image quality and consequently the percentage of the feasibility of the measurements will go down. In our analysis we have done only partial observations about diastolic data since most of the video clips were recorded at a frame rate lower than 60 fps (between 40 to 60 fps) and this probably would have resulted in an underestimation of the peak values of those rapid events. The feasibility of these measurements was less in short axis views than in the long axis views: this could partially be consequent to the movements of the whole left ventricle in directions others than radial or circumferential, partially to the quality of the images and also to the tracking accuracy of the algorithm in short axis.

The first issue is an intrinsic limitation of the 2D approach and it will probably be solved when the system will be applied to 3D echocardiography. On the other hand, rapid improvement in technology in terms of improved tracking of the algorithm and better lateral resolution of images even with higher image fps will enhance the results.

The assessment for longitudinal systolic ε and SR was 100% in apical 4C views, but it fell to 73% for ε and 64% for SR in apical 2C view. This was mainly due to the poor lateral resolution in the apical anterior segment, which is the most difficult segment to demark with an endocardial border.

Our results are very encouraging if we consider that in other studies (where 4C video-clips were analyzed with speckle tracking technology) 20% of the segments couldn't be processed [13]. Reference values derived in our study for longitudinal systolic velocity are similar to those reported using TDI and/or MRI [13,18-20] and confirm that the longitudinal velocity decreases from base to apex. The significant increase of longitudinal ε and SR from base to apex observed in our study has not been detected with TDI [11] but has been reported with tagged MRI.

2D strain technologies have demonstrated their value in several clinical conditions and now that they have been shown to be highly accurate and reproducible, they are going to be applied in the large cardiac disease population, substituting the TDI technology that gives consistent but limited information in respect to 2D based technology.

Validation against MRI is recommended for all imaging technology and is widely considered to be the gold standard because it allows the measurement all three velocity vectors and thus analyzes regional strain in three dimensions; but the peak values obtained with MRI must also be considered carefully because this technique has a lower temporal resolution compared to echocardiography, and, with 30 fps it could underestimate both diastolic and systolic values, especially when heart rate is rapid, as occurs in children [21,22].

Having derived the normal values for pediatric and young adult population it is now important to open up this exciting new field of basic science to study physiology of the ventricular function and its change with the human growth. This will also be very useful in the clinical assessment of segmental and global ventricular function in congenital heart disease where other echocardiographic parameters are often less applicable.

## Conclusion

The major limitation of this study was that we were restricted in considering only the data for systolic events because the feasibility for diastolic events was consistently reduced, especially in short axis view. This was due to sub-optimal tracking followed by the algorithm but rapid evolution of ultrasound technology and improvement in software engineering will rapidly overcome this limitation.

2D based tissue tracking system is highly reliable and applicable for the study of systolic events in adults and children. Reference values for children and adults have been derived for various segments of myocardium and significant difference between adults and children in longitudinal and circumferential Strain and SR has been demonstrated. This difference is more evident in the circumferential strain of the apical segments.

The capability of this system to analyze curved segments as well as rectilinear segments consistently may provide valuable insights into physiology and patho-physiology of left ventricular function [23]. It will offer new possibilities for quantification of echocardiographic studies, which, until now, have been based mostly on calculation of volumes, thickness and blood flow analysis. With this new approach a direct quantification of segmental and global myocardial function will be feasible. To obtain this complete evaluation in the everyday practice, the echocardiographic laboratories will need dedicated infrastructure for post-processing [24].

## Competing interests

CB acted as consultant to ESAOTE in the development of the clinical software.

## Authors' contributions

CB: Conceptualized and designed the study, executed all the echocardiographic exams, analyzed and interpreted the data, overviewed at the file processing and wrote the manuscript. AM: Reviewed the manuscript and made substantial contribution to the conception and design of the study. MDD: Reviewed the manuscript, made substantial contribution of data interpretation, contributed to the statistical analysis. BdeC: Contributed to drafting of the manuscript and statistical analysis. DN: Examined clinically all the subjects and participated in the post processing of echocardiographic files. EDA: Participated in the post processing of echocardiographic files and overviewed all data processing and collection. EL: Reviewed the manuscript for relevant intellectual content. MCH: Made substantial contribution to study conception and design. JSD: Reviewed the manuscript for relevant intellectual content. CA: Participated in the post processing of echocardiographic files and overviewed all data processing and collection. FM: Made substantial contribution to study conception and design. MC: Made substantial contribution to study conception and design.
